# Liquid Application Dosing Alters the Physiology of Air-Liquid Interface Primary Bronchial Epithelial Cultures and In vitro Testing Relevant Endpoints

**DOI:** 10.21203/rs.3.rs-2570280/v1

**Published:** 2023-02-24

**Authors:** Nicholas M. Mallek, Elizabeth M. Martin, Lisa A. Dailey, Shaun D. McCullough

**Affiliations:** University of North Carolina at Chapel Hill; National Institute of Environmental Health Sciences; Environmental Protection Agency; Environmental Protection Agency

**Keywords:** in vitro to in vivo extrapolation (IVIVE), new approach methods (NAMs), inhalation risk assessment, air-liquid interface (ALI)

## Abstract

Differentiated Primary human bronchial epithelial cell (dpHBEC) cultures grown under air-liquid interface (ALI) conditions exhibit key features of the human respiratory tract and are thus critical for respiratory research as well as efficacy and toxicity testing of inhaled substances (*e.g*., consumer products, industrial chemicals, and pharmaceuticals). Many inhalable substances (*e.g*., particles, aerosols, hydrophobic substances, reactive substances) have physiochemical properties that challenge their evaluation under ALI conditions *in vitro*. Evaluation of the effects of these methodologically challenging chemicals (MCCs) *in vitro* is typically conducted by “liquid application,” involving the direct application of a solution containing the test substance to the apical, air-exposed surface of dpHBEC-ALI cultures. We report that the application of liquid to the apical surface of a dpHBEC-ALI co-culture model results in significant reprogramming of the dpHBEC transcriptome and biological pathway activity, alternative regulation of cellular signaling pathways, increased secretion of pro-inflammatory cytokines and growth factors, and decreased epithelial barrier integrity. Given the prevalence of liquid application in the delivery of test substances to ALI systems, understanding its effects provides critical infrastructure for the use of *in vitro* systems in respiratory research as well as in the safety and efficacy testing of inhalable substances.

## Introduction

Exposure to a broad range of inhalable substances including inhaled pharmaceuticals, consumer products, industrial chemicals, and ambient air pollutants influences human health worldwide. Inhalation is one of the three primary routes of chemical and pharmaceutical exposure and many inhaled substances elicit their effects, beneficial or adverse, in the respiratory tract, which serves as the portal of entry. The vast number of data-poor new and existing inhalable substances, including ambient or occupational exposures, pharmaceuticals, and consumer and commercial products, as well as mixtures of different substances, and repeated exposure scenarios precludes the feasibility of using traditional *in vivo* animal studies to evaluate the potential adverse effects of these exposures and scenarios^1^. The 2007 National Research Council report, *Toxicity Testing in the 21st Century: A Vision and a Strategy*, outlined a strategy to address these limitations through the use of *in vitro* and computational new approach methodologies (NAMs) to advance the throughput and human relevance of toxicity testing. These recommendations have been used by regulatory agencies, including the US Environmental Protection Agency, to develop strategic plans to increase the use of NAMs to generate data for decision making^2,3^.

The respiratory tract epithelium functions as a physical barrier between inhaled materials and underlying tissues. In the nasal and tracheobronchial airways, the secretion of mucus and presence of beating cilia also function as a mucociliary escalator to facilitate clearance of inhaled materials and pathogens. Further, respiratory epithelial cells participate in the induction and regulation of the immune response to injury and inhaled insults through the secretion of pro-inflammatory cytokines. Primary human airway epithelial cell cultures that have been differentiated and subsequently maintained under ALI conditions exhibit key features of their respective regions of the human respiratory tract *in vivo* including the formation of a selectively permeable and electrically resistant barrier, the presence of beating cilia, release of pro-inflammatory cytokines, and mucus secretion^4–8^. ALI-differentiated primary human bronchial epithelial cell (dpHBEC-ALI) and primary human nasal epithelial cell (dpHNEC-ALI) systems have been widely used to model their respective regions of the respiratory tract to advance our understanding of respiratory tract development and disease. They are also a critical resource in support of efforts to increase the *in vivo* human relevance of inhaled test substance evaluation^9–12^.

Evaluating the effects of inhaled gases and vapors on differentiated primary airway epithelial cell culture models while maintaining ALI conditions can generally be accomplished using existing *in vitro* exposure apparatus/technology. In contrast, many other types of test substances (*e.g*., particles, aerosols, hydrophobic substances, reactive substances, biomolecules, et cetera) have physical and/or chemical properties that pose challenges to their evaluation under ALI conditions. The evaluation of these inhaled methodologically challenging chemicals (MCCs) using *in vitro* systems has often relied on the application of test substances in aqueous solutions/suspensions to the apical surface of ALI models of the respiratory tract (described here as “liquid application”, but also referred to as “direct application” or “liquid dosing” elsewhere). Further, liquid application dosing is often used to deliver exogenous stimuli (*e.g*., lipopolysaccharide (LPS), tumor necrosis factor alpha (TNF-a) or small molecules to ALI cultures for basic science research and pharmaceutical development, respectively.

When using liquid application dosing, the effect of the test substance treatment is typically assessed by comparison to the respective aqueous solvent (*e.g*., water, saline, buffered saline, cell culture medium, *et cetera*) as a vehicle control. The application of exogenous liquid alone (*i.e*., in the absence of a test substance) disrupts ALI conditions, a key physiologically relevant feature of these culture models; however, the effects of applying liquid to differentiated ALI cultures are typically not considered in the analysis and interpretation of study data. Further, while liquid application dosing is commonly used for the delivery of test substances and biological stimuli in ALI systems in inhalation toxicology research and testing, the effects of applying liquid to ALI cultures have not been well characterized. Thus, the potential for liquid application dosing to affect the physiological relevance of the differentiated ALI *in vitro* system, as well as confound test substance exposure effects and the extrapolation of study data to human health outcomes are poorly understood. Addressing this knowledge gap by determining whether disrupting the *in vivo*-relevant ALI conditions alters dpHBEC culture physiology is critical to providing context for the use of liquid application dosing of *in vitro* ALI systems for respiratory biology, inhalation toxicology, pharmaceutical development, and disease research. Here, we report the results of a study describing the effects of the application of liquid, in the absence of a test substance, to a dpHBEC-ALI co-culture model on endpoints that are relevant to *in vivo* bronchial epithelial tissue physiology. These endpoints included global transcriptional regulation, biological pathway activity, ciliary beat frequency, regulation of stress-responsive cellular signaling pathways, release of pro-inflammatory cytokines and growth factors, and epithelial barrier function.

## Methods

### Differentiated pHBEC co-culture ALI model

**Figure 1:**
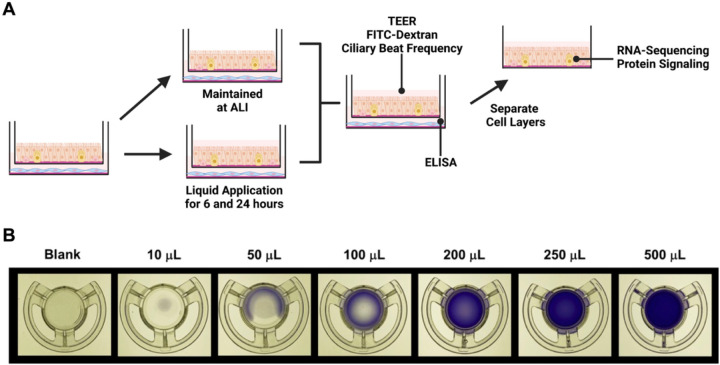
Experimental design. (A) IMR90 fibroblasts were seeded onto collagen-coated tissue culture wells 24 hours before being combined with dpHBEC under ALI conditions. 24 hours after the cells were combined, 250 μL of liquid (ALI medium) was applied to the apical surface of the dpHBECs for either 6 or 24 hours. A matched set of cultures was maintained under ALI conditions to serve as the control. The indicated endpoints were evaluated to determine the effect of the application of liquid on dpHBEC cultures. (B) Titration of apical volumes of crystal violet solution in 12 mm Transwell inserts used to visualize the relative coverage of the insert surface area at different applied liquid volumes. The 250 μL volume was determined to provide the best balance between the smallest volume and uniformity of liquid coverage.

pHBEC were obtained via bronchial brushing from healthy, donors ages 18–40 that were not currently smoking and had no more than a one pack-year of lifetime smoking history. Donors gave their consent after being informed of risks and procedures. The consent and collection protocol were approved by the UNC School of Medicine Committee on the Protection of the Rights of Human Subjects and by the US EPA. Collection of pHBEC from volunteers was performed in accordance with relevant guidelines and regulations. pHBEC were isolated from brush biopsy (“passage 0”) and expanded to passage 3 prior to being plated on 12 mm Transwell^®^ inserts (Corning #3460; 0.4 μm pore polyester membranes), becoming confluent, and differentiating for 24 days under ALI conditions. Detailed descriptions of the techniques, reagents, and materials used for the culture/expansion of pHBEC and differentiation of pHBEC at ALI used in this study are available as open access methods documents^13^. Day 24 ALI cultures were visually evaluated for the presence of beating cilia and the production of mucus as indicators of differentiation status to qualify their use in experiments. Donor demographics are listed in Supplementary Table 1. All cultures were at ALI day 24 at the beginning of experiments. The human lung fibroblast cell line IMR90^14^ was obtained from the American Type Culture Collection (ATCC, No. CCL-186, Batch #64155514), and cultured as described in detail in the open-access methods document^15^. Short tandem repeat (STR) service provided by ATCC (cat #135-XV) was used to authenticate IMR90 cells (Supplementary Fig. 1). As described in Fig. 1A, IMR90 fibroblasts were seeded onto a collagen-coated 12-well plate (Corning, #3513) at a density of 1×10^5^ cells/mL in 800 μL of Pneumacult ALI medium (StemCell Technologies, #05001). The following day, day 23 dpHBEC-ALI inserts were added into the fibroblast-seeded wells (dpHBEC-IMR90 ALI model). The dpHBEC-IMR90 ALI model was used for experiments on the following day. The dpHBEC-IMR90 ALI model was maintained at ALI or subjected to the addition of 250 μL (223 μL/cm^2^; equal to 73.5 μL in a 6.5 mm Transwell insert) of ALI medium to the apical surface of the culture (“liquid application”). This was the smallest volume of ALI medium that resulted in the most uniform coverage of the dpHBEC cultures during the 24-hour treatment duration as determined by a titration of apical volumes of a 0.5% Crystal Violet (Sigma, #C6158–50G; dissolved in Dulbecco’s Phosphate Buffered Saline (Gibco #14190–144)) stain solution to 12 mm Transwell inserts (Fig. 1B). Inserts were placed on a white light transilluminator (FUJIFILM #IPE4046) and photographed with an iPhone SE (Apple, Model #MX9M2LL/A). The cells were kept submerged in ALI medium for either 6 or 24 hours to represent “early” and “late” effects, respectively. Additionally, the 24-hour treatment is consistent with the liquid application treatment duration utilized in the recent OECD case study on the use of an ALI-differentiated *in vitro* NAM for an IATA to refine inhalation risk assessment for point of contact toxicity^12,16–18^. The effect of liquid application on global gene expression, stress-responsive signaling protein phosphorylation, growth factor secretion, pro-inflammatory cytokine secretion, trans-epithelial electrical resistance (TEER), and permeability to a 20 kDa fluorescent dextran was then determined immediately after 6 and 24 hours of liquid application (Fig. 1A).

### RNA Isolation

Total RNA was isolated using the PureLink RNA Mini kit (ThermoFisher #12183025) according to the detailed open access method^19^. Briefly, dpHBEC samples were harvested by the addition of 250 μL RNA lysis buffer containing 1% b-mercaptoethanol, triturated, and transferred to Eppendorf tubes on ice prior to storage at −80°C until extraction. Total RNA was eluted in nuclease-free water (ThermoFisher, #AM9937) following extraction and column purification. Total RNA was quantified using a NanoDrop OneC UV-Vis Spectrophotometer. Purified RNA was stored at −80°C until submission for RNA-Sequencing or preparation of cDNA for quantitative real-time polymerase chain reaction (qRT-PCR).

### Sequencing, DESeq2, and qRT-PCR Validation

Library preparation and sequencing were performed by the University of North Carolina at Chapel Hill High Throughput Genomic Sequencing Facility. Briefly, 100–500 ng of RNA were used to make libraries using a KAPA Stranded mRNA-Seq Kit, with KAPA mRNA Capture Beads (Roche Sequencing and Life Science #07962207001). Libraries underwent quality control via dsDNA High Sensitivity Qubit following cDNA synthesis and adaptor ligation (Q32851). Libraries were normalized to 5 nM and pooled in equal amounts and then processed on a miSeq nano flowcell (MS-103–1001). Samples were run on an Illumina Novaseq 6000 (Illumina, San Diego, CA), and calibrated against 1% PhiX as a positive control during sequencing. Base call files were transformed into FASTQ files using bcl2fastq software (version 2.20.0). Mapping of sequence reads to the human genome (GRCh38) was performed by STAR (version 2.5) with the following parameters: -- outSAMattrIHstart 0 --outFilterType BySJout --alignSJoverhangMin 8 -- outMultimapperOrder Random (other parameters at default settings). Mapped read counts per gene were collected by Subread feature Counts (version 1.5.0–0-p1). Genes with a minimum average of normalized mapped read counts > 50 in at least one category were selected for differential gene expression analysis. Differentially expressed genes (DEGs) were identified by DESeq2 (v 3.13) using filters of FDR < 0.01. DEG results are available to the public on the NCBI Gene Expression Omnibus (series number: GSE198884). Fold change expression values derived from RNA-sequencing-derived DESeq2 were validated against standard real-time qRT-PCR methods. Briefly, cDNA was synthesized using the iScript Reverse Transcription Kit (Bio-Rad, #1708891) according to the manufacturer’s protocol. Fold change values were assessed via primers and hydrolysis probes (Supplementary Table 2) on a CFX96 Touch (Bio-Rad). Target gene C_q_ values were normalized to β-Actin and expressed as fold changes between liquid application and ALI conditions using the Pfaffl method^20^.

### Ingenuity Pathway Analysis

The number of statistically significant alternatively regulated genes identified in the 24-hour liquid application treatment (10,239 genes) exceeded the maximum number of genes (8,000) that can be analyzed by the IPA software. Thus, we generated a sub-set of each DEG list that included only statistically significant genes that were alternatively regulated with an absolute value log_2_ fold change > 2.0. These “pathway analysis” gene sets were uploaded to Ingenuity Pathway Analysis software (Version 65367011). Gene lists were exported for each of the top 10 canonical pathways as determined by −log(*p*-value). The 10 genes with the greatest absolute fold change value were exported as tables for the top 10 most significant canonical pathways.

### Protein Collection and Immunoblotting

Total protein was isolated via a RIPA extraction. Briefly, dpHBEC samples were harvested by the addition of 250 μL of RIPA lysis buffer (50 mM tris-base, pH 8.0, 150 mM NaCl, 400 uM EDTA, 10% glycerol, 1% Triton-X, 0.1% SDS, 0.1% sodium deoxycholate, with 1X protease inhibitor (Sigma, #11697498001) and phosphatase inhibitor (Sigma, #04906837001) cocktails) to each insert, scraping with a wide-bore P200 pipette tip (USA Scientific, #10118410), and transfer to an Eppendorf tube prior to incubation on ice for 20 minutes. Samples were centrifuged at 13,000 × *g* for 15 minutes to pellet cell debris and supernatants were transferred to new Eppendorf tubes on ice. Three aliquots (10 μL) were removed from each sample for determination of protein concentration by BCA assay. 5X Laemmli buffer (120 mM Tris, pH 6.8, 400 mM dithiothreitol, 20% glycerol, 4% SDS, 0.025% bromophenol blue) was then added to the remaining sample volume before the samples were incubated at 95°C for 5 minutes. Samples were stored at −80°C until used for immunoblotting. The antibodies and dilutions used for immunoblotting are provided in Supplementary Table 3. All samples were transferred on 0.45 μM nitrocellulose membranes (Bio-Rad, #1620115) in transfer buffer (48 mM tris-base, 39 mM glycine, 20% methanol). Chemiluminescence was generated via a horseradish peroxidase-conjugated secondary antibody (Jackson ImmunoResearch, #711-036-152) and Clarity Western ECL Blotting Substrate (Bio-Rad #1705060). Images were taken on a ChemiDoc MP Imaging System (Bio-Rad), and densitometry was generated using Bio-Rad Image Lab Software (Version 6.1.0, Build 7).

### Evaluation of growth factors & pro-inflammatory cytokine secretion

Conditioned medium was collected from the basolateral compartment of dpHBEC-ALI cultures immediately before liquid application, and 6 or 24 hours after liquid application. Conditioned media were centrifuged and syringe filtered through 0.22 μm pore PVDF membranes (Millipore Sigma, #SLGV033RS) to remove cellular debris before storage at −80°C until use. Conditioned media were probed for cytokine secretion by the V-PLEX Human Proinflammatory Panel II (4-Plex) (Mesoscale Discovery, K15053D-1) and growth factor secretion by the U-PLEX Development Pack (Mesoscale Discovery, K15228N-1) according to the manufacturer’s protocols. Briefly, conditioned media samples were diluted two-fold, and assayed in triplicate with either four 10-fold serial dilutions (for cytokines) or four two-fold serial dilutions (for growth factors) to determine the limits of detection in our dpHBEC-ALI cultures. The MSD plate was washed thrice with 150 μL of wash buffer per well prior to the addition of 50 μL of diluted sample to individual wells and incubation with shaking (300 RPM) for two hours at room temperature. Samples were then aspirated and the plate was washed thrice with 150 μL of wash buffer prior to the addition of 25 μL of detection antibody solution to each well and incubation with shaking (300 RPM) for two hours at room temperature. The antibody solution was then aspirated and the plate was washed thrice with 150 μL wash buffer before the addition of 150 μL of 2X Read Buffer T to each well. The sample plate was read on the Meso Quickplex SQ 120, and analyte concentrations were determined using the MSD Discovery Workbench software (Version 4.0.12)

### Trans-Epithelial Electrical Resistance

Trans-epithelial electrical resistance (TEER) was measured using the EVOM2 (World Precision Instruments) with EndOhm cup for 12 mm inserts (World Precision Instruments) at the indicated time points as described in a detailed open-access methods document^21^. Briefly, resistance measurements were obtained for both experimental samples and insert only (without cells). TEER values from insert only samples were subtracted from experimental sample resistance values and the resulting values were then multiplied by the insert surface area to calculate values in Ω × cm^2^^22^. Three cultures were used for each condition per experiment and each culture was assayed in technical triplicate. The mean (±SD) of three independent experiments is shown. TEER was treated as a destructive assay and no additional assays were conducted on cultures that were analyzed by TEER.

### Fluorescein Isothiocyanate-labeled Dextran Assay

The translocation of a 20 kDa fluorescein isothiocyanate (FITC)-labeled dextran (Sigma, FD20) was measured as described in a detailed open-access methods document^23^. Briefly, experimental sample inserts and insert only (without cells) were transferred to a new multi-well plate containing 800 μL of phenol-red free MEM (ThermoFisher, #51200038) in each well. The apical medium was carefully aspirated from cultures after liquid application for 6 or 24 hours and 250 μL of a 1 mg/mL suspension of FITC-dextran in Pneumacult ALI medium (StemCell Technologies, #05001) was added to the apical compartment of each insert. Samples were incubated in the dark for 20 minutes at 37°C, and the basolateral media containing translocated FITC-dextran suspension was collected and transferred to a 96-well clear bottom microplate (Corning, #3904) prior to determination of the fluorescence of each sample at 490/520 nm Ex/Em on a ClarioStar Plus plate reader. Three cultures were used for each condition per experiment and each culture was assayed in technical triplicate. The mean (±SD) of three independent experiments is shown. The FITC-dextran assay was considered to be destructive and no additional assays/endpoints were conducted/evaluated on cultures after this assay.

### Statistical Analysis

Statistical analyses for protein densitometry, cytokine and growth factor secretion, TEER, FITC-dextran, ciliary beat frequency and total ciliated area, and viability were conducted using GraphPad Prism 9.0.1 (GraphPad Software). The statistical significance of differences between each duration of liquid application and untreated controls were evaluated using a parametric unpaired t-test. A *p* value of ≤0.05 was considered statistically significant for all analyses.

## Results

### Liquid application alternatively regulates global gene expression in dpHBEC-ALI

**Figure 2:**
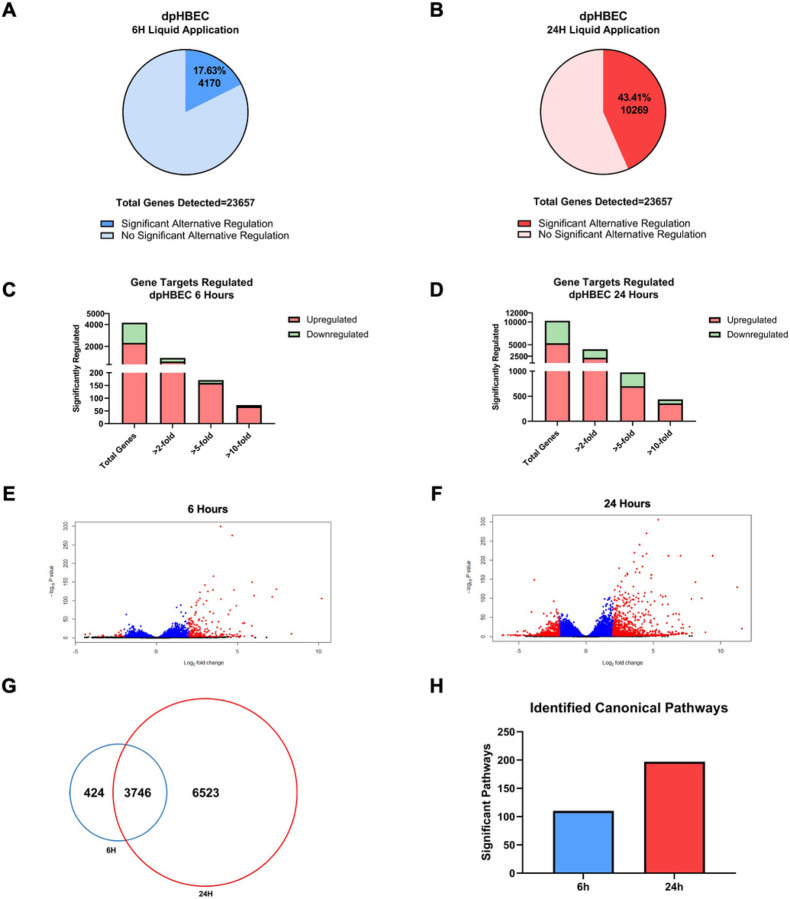
Global transcriptome changes following liquid application exposure for 6 or 24 hours. (A-B) The total amount of significantly alternatively regulated genes (adjusted *p*-value <0.05) after 6 hours (A) or 24 hours (B) of liquid application, respectively. (C-D) The total number of significant alternatively regulated genes from (A-B) separated into fold change expression thresholds after 6 hours (C) or 24 hours (D) of liquid application, respectively. (E-F) Volcano plots of the gene datasets described in (A-B). Blue points have an adjusted *p*-value of <0.01, and red points have an adjusted *p*-value of <0.05 and a log_2_(foldchange) >1. (G) Venn Diagram comparison displaying the number of significant (adjusted *p*-value <0.05) unique and shared genes between the 6 and 24 hour liquid application dataset described in (A-B). (H) Total number of significant canonical pathways regulated after liquid application for 6 or 24 hours according to Ingenuity pathway analysis of the dataset from (A-B).

Following ALI differentiation, the transcriptional profiles of dpHBEC cultures recapitulate those of their corresponding *in vivo* tissue^4–6^. We began our assessment of the impact of liquid application on dpHBEC-ALI cultures with the evaluation of its effect on global transcriptional programming using RNA-sequencing. Of the tested transcripts (*n* = 33,121 in the RNA-seq Data set), 23,657 (71%) were detected in dpHBECs. The application of liquid to dpHBEC-ALI cultures for 6 and 24 hours resulted in the significant (adjusted *p*≤0.05) alternative expression of 4,170 and 10,269 genes, respectively (Fig. 2A and 2B). Of the 4,170 genes that were alternatively expressed at 6 hours of liquid application, 626 and 326 were up- and down-regulated greater than 2-fold, 160 and 11 were up-and down-regulated greater than 5-fold, and 68 and 5 were up- and down-regulated greater than 10-fold, respectively (Fig. 2C). Of the 10,269 genes that were alternatively expressed at 24 hours of liquid application, 2,159 and 1,865 were up- and down-regulated greater than 2-fold, 700 and 274 were up- and down-regulated greater than 5-fold, and 358 and 79 were up- and down-regulated greater than 10-fold, respectively (Fig. 2D). The 6- and 24-hour time points had 424 and 6,523 unique alternatively expressed genes, respectively, and 3,746 alternatively expressed genes in common (Fig. 2G). A complete description of the DEG sets is available through the NCBI Gene Expression Omnibus (series # GSE198884).

**Figure 3:**
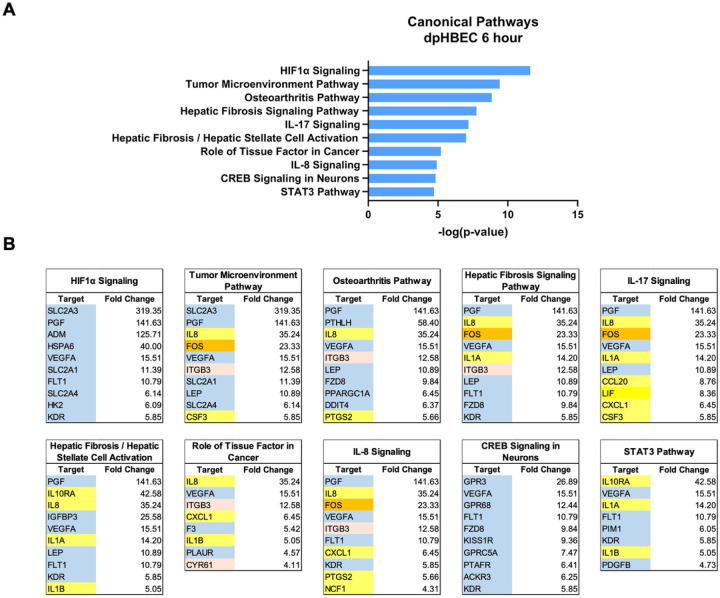
Ingenuity pathway analysis of alternatively regulated genes after 6 hours of liquid application on dpHBEC-ALI cultures. (A) The top 10 most significant canonical pathways by the −log(*p*-value) identified after 6 hours of liquid application. (B) The top 10 genes, by absolute value, in each canonical pathway identified in (A), and their respective fold change expression over ALI cultures. Only genes exhibiting a log_2_ fold change 2.0 were used in this analysis.

**Figure 4:**
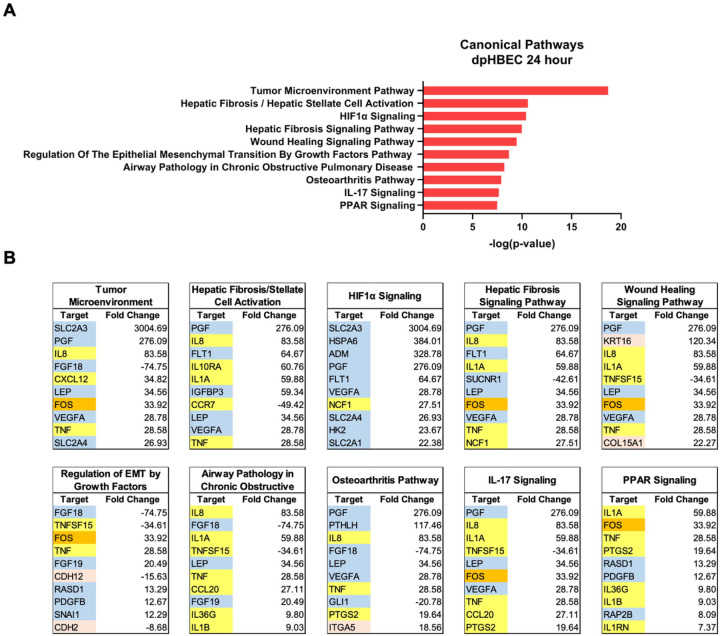
Ingenuity pathway analysis after 24 hours of liquid application on dpHBEC-ALI cultures. (A) The top 10 most significant canonical pathways by the −log(*p*-value) identified after 24 hours of liquid application. (B) The top 10 genes, by absolute value, in each canonical pathway identified in (A), and their respective fold change expression over ALI cultures. Only genes exhibiting a log_2_ fold change 2.0 were used in this analysis.

Ingenuity Pathway Analysis of significantly alternatively expressed genes indicated that liquid application resulted in the significant alternative regulation of 110 and 197 canonical pathways in pHBEC-ALI cultures at 6 and 24 hours, respectively (Fig. 2H). The 10 most significant pathways (by −log(*p*-value)) identified at 6 and 24 hours are listed in Figs. 3A and 4A and the 10 genes with the largest magnitude of fold change expression, by absolute value, for each of the top alternatively regulated pathways are listed in Figs. 3B and 4B, respectively. Several alternatively expressed genes were common to the 10 most significantly alternatively regulated pathways following 6 hours of liquid application, including: the growth factors placental growth factor (PGF, 141.6-fold) and vascular endothelial growth factor A (VEGFA, 15.5-fold) and pro-inflammatory mediators such as interleukin (IL)-8 (IL-8, 35.2-fold), IL-1α (IL-1α, 14.2-fold), IL-1β (IL-1β, 5.1-fold), and cyclooxygenase-2 (COX-2 5.7-fold). Many alternatively expressed genes were also common to the alternatively regulated pathways after 24 hours of liquid application including: the growth factors PGF (276.1-fold) and VEGFA (28.9-fold), and the pro-inflammatory mediators IL-8 (83.6-fold), IL-1α (59.9-fold), IL-1β (9.0-fold), and PTGS2 (19.6-fold). Additional highly expressed genes at 24 hours included platelet derived growth factor subunit B (PDGF-B, 12.7-fold), and tumor necrosis factor (TNF, 28.6-fold). The alternative expression of selected targets identified in the RNA-seq analysis (IL-8, IL-1α, and COX-2) were validated by qRT-PCR (Supplemental Fig. 2).

#### Liquid application exposure of dpHBEC-ALI increases HIF-1α protein levels and the phosphorylation of ERK and p38 cellular signaling pathways

**Figure 5:**
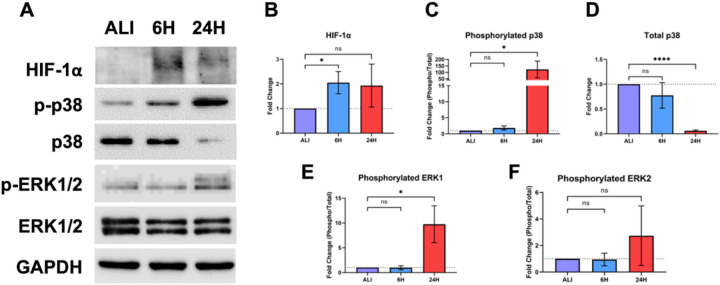
Liquid application modulates stress-responsive protein signaling pathways in dpHBECs. (A) Representative images of protein signaling western blots for levels of phosphorylated and total p38, HIF-1α, phosphorylated and total ERK1/2, and GAPDH loading control. Representative images represent n=3 biological replicates. Densitometry showing (B) changes in total p38, (C) the ratio of phosphorylated to total p38, (D) the induction of HIF-1α, (E) the ratio of phosphorylated to total ERK1, and (F) the ratio of phosphorylated to total ERK2 normalized to GAPDH loading control. Data represent the mean of n=3 biological replicates and error bars represent SD. *, ****, and ns indicate p ≤ 0.05, p≤0.0001, and not significant, respectively.

HIF-1 is a heterodimeric transcription factor that regulates the expression of genes involved in the cellular response to hypoxia, as well as tissue vascularization and the maintenance of tissue homeostasis^24,25^. HIF-1 activation is regulated by the stabilization of the HIF-1α subunit, which occurs in response to hypoxic conditions^24^. The application of liquid to an ALI culture reduces exposure of the apical culture surface to oxygen. Thus, we evaluated whether liquid application resulted in the stabilization of HIF-1α, the regulatory subunit of HIF-1, which was significantly increased (2.05-fold (±0.45)) at 6, but not 24, hours of liquid application (Fig. 5B).

The ERK and p38 mitogen activated protein kinase (MAPK) signaling pathways regulate the response of the respiratory epithelium to oxidative, inflammatory, and pathogenic stimuli^26–29^. Hyperactivation of these pathways also leads to respiratory disease including asthma, COPD, lung injury, and lung cancer^30–31^. Given their role in mediating homeostasis in the respiratory tract, we sought to determine whether these signaling pathways they would be alternatively regulated in response to liquid application (Fig. 5A). We observed a significant 122.9-fold (±64.56) increase in p38 MAPK phosphorylation that was accompanied by an 16.5-fold (±3.75) decrease in total p38 protein levels at 24 hours of liquid application exposure (Fig. 5C and D). Phosphorylation levels of ERK1 were significantly increased after 24 hours of liquid application exposure, whereas ERK2 phosphorylation levels were unchanged (Figs. 5E and F).

### Liquid application increases pro-inflammatory cytokine and growth factor secretion in dpHBEC cultures

**Figure 6:**
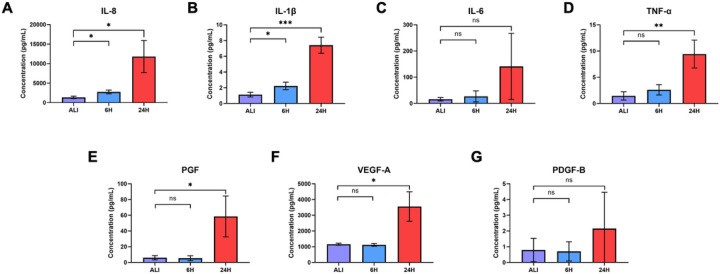
Liquid application increases the release of pro-inflammatory cytokines and growth factors. Effects of 6- and 24-hour liquid application on the amount of (A) IL-8, (B) IL-1b, (C) IL-6, (D) TNF-a, (E) PGF, (F) VEGF-A, and (G) PDGF-B detected in the basolateral medium. Data represent the mean of *n*=3 biological replicates and error bars indicate SD. *, **, ***, and ns indicate *p* 0.05, *p* 0.01, *p* 0.001, and not significant, respectively.

Bronchial epithelial cells play a key role in orchestrating inflammation within the respiratory tract in response to inhaled toxicants and other cellular stressors through the secretion of pro-inflammatory cytokines^32^. After observing the upregulation of pro-inflammatory cytokine transcripts at both 6 and 24 hours of liquid application, we sought to determine whether these changes also resulted in the increased release of the corresponding pro-inflammatory cytokine proteins. We observed significant increases in the secretion of IL-8, IL-1β, and TNFα into the basolateral medium at both 6 and 24 hours of liquid application exposure (Fig. 6A, B, and D). While IL-6 secretion exhibited an upward trend after 24 hours of liquid application exposure, the change was not statistically significant (Fig. 6C). Cytokine concentration and fold change values are reported in Table 1.

The regulation of growth factor signaling plays an important role normal airway homeostasis. Aberrant growth factor signaling occurs in response to chemical exposures and plays a role in airway disease^33–36^. Liquid application resulted in the upregulation of several growth factor transcripts at 6 and 24 hours so we sought to determine whether changes translated to increased growth factor secretion. We observed significant increases in the secretion of both PGF and VEGF-A, but not PDGF-B, into the basolateral medium after 24 hours of liquid exposure (Fig. 6E–G). Growth factor concentrations and fold change values are reported in Table 1.

### Liquid application exposure of dpHBEC-ALI decreases epithelial barrier function but not ciliary function

**Figure 7:**
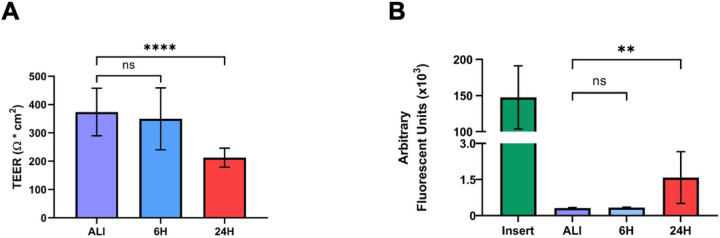
Liquid application reduces epithelial barrier integrity of dpHBEC cultures. (A) Effects of 6- and 24-hour liquid application on TEER of dpHBEC layer. (B) Effects of 6- and 24-hour liquid application on permeability of the dpHBEC layer to the translocation of 20 kDa FITC-dextran. Data represent the mean of n=3 biological replicates and error bars represent SD. ****, **, and ns indicate p≤0.0001, p≤0.01, and not significant, respectively.

*In vivo*, the normal bronchial epithelium functions in host defense as a physical barrier and a mucociliary escalator. In its role as a barrier, the normal bronchial epithelium forms an electrically resistant tissue layer that is selectively permeable to small molecules that separates inhaled substances from underlying host tissue^37^. While often affected by exposure to inhaled insults, loss of bronchial epithelial barrier integrity is also a hallmark of both acute and chronic respiratory disease^38–41^. Further, loss of epithelial barrier integrity also results in the loss of apical-basolateral polarization and is a key aspect of epithelial-to-mesenchymal transition (EMT), an early step in epithelial carcinogenesis^42^. We evaluated the effect of liquid application on epithelial barrier integrity by determining the electrical resistance of the epithelial layer by trans-epithelial electrical resistance (TEER). Cultures maintained at ALI exhibited a mean TEER of 373.7 (±83.9) Ω × cm^2^, which was significantly reduced to 212.7 (±33.3) Ω × cm^2^ after 24 hours of after liquid application (Fig. 7A); however, TEER was not significantly affected after 6 hours of liquid application (349.7 ± 109.2 Ω × cm^2^). We then evaluated whether the decreased electrical resistance coincided with an increase in the small molecule permeability of the epithelial barrier. In concordance with the observation of decreased TEER, liquid application significantly increased the permeability of the epithelial layer to 20kDa FITC-conjugated dextran, which is a similar molecular weight to small biomolecules (*e.g*., IL-6), after 24 hours (1581 ± 1076 arbitrary fluorescent units (AFU)), but not at 6 hours (329.9 ± 19.6 AFU), when compared to cultures maintained at ALI (310.0 ± 21.9 AFU) (Fig. 7B).

The normal *in vivo* bronchial epithelium contains actively beating cilia, a biological feature that is recapitulated *in vitro* by dpHBEC cultures. Despite observing the effects on barrier integrity described above, we did not observe any significant effect of liquid application on CBF or the percentage of active ciliated surface area of dpHBEC cultures (Supplementary Fig. 3).

## Discussion

The direct application of experimental treatments in aqueous solvents to differentiated primary human airway epithelial cell cultures is, and has been, a common practice for the dosing of *in vitro* ALI systems with biological stimuli, experimental interventions, and other test substances. Liquid application dosing is considered to be practical due to its expediency, low cost, and minimal technical requirements. The application of liquid to the apical surface of differentiated primary human airway epithelial cell cultures disrupts ALI conditions that are a critical component of their ability to represent the luminal surface of the respiratory tract *in vivo*; however, the effects of different liquid application conditions (*e.g*., liquid volume, type of solvent, and treatment duration) are poorly understood. The success of initiatives to leverage *in vitro* test systems in respiratory biology, pharmaceutical development, disease research, and inhaled chemical testing depends on building confidence in their ability to represent *in vivo* human-relevant physiology. Additionally, characterizing the effects of liquid application as a dosing modality for ALI-dpHBEC cultures is critical to creating context for the interpretation and integration of data collected with this method into applicable decision making processes.

### The application of liquid to dpHBEC causes a multi-disease-like phenotype that is characterized by effects that are similar to those induced by inhaled toxicants

The use of differentiated primary airway epithelial cell systems as an *in vitro* model with enhanced physiological relevance compared to either their undifferentiated counterparts or analogous cell lines is predicated on the assumption that the cultures are comparable to their donor tissue of origin. That is, cultures generated from “normal healthy” donors are representative of the respective region of the respiratory tract *in vivo* in the “normal healthy” population. Here we report that the application of liquid to dpHBEC caused significant transcriptional reprogramming of 4,169 and 10,268 genes, which represented 17.6% and 43.4% of expressed genes, at 6 and 24 hours of liquid application, respectively (Fig. 2). By comparison, the number of genes that were significantly alternatively regulated by 24 hours of liquid application were more than four- and eight-fold greater than the number of genes that were significantly alternatively regulated between confluent pHBEC grown in submerged culture and pHBEC following 24- or 28-day differentiation at ALI^7,43^. Pathway analysis of transcriptional changes indicated that the application of liquid to dpHBEC cultures caused effects that were consistent with cancer/epithelial-to-mesenchymal transition (EMT), fibrosis, hypoxia, wound healing, and pro-inflammation (Figs. 3 and 4). Analysis of the RNA-sequencing data indicated that the transcriptional profiles of cultures following liquid application were no longer consistent with their “normal healthy” counterparts (*i.e*., incubator controls maintained at ALI). Thus, we then evaluated the effect of liquid application on endpoints that are involved in bronchial epithelial homeostasis and normal tissue function. Our observations demonstrate that the application of liquid alone caused dpHBEC cultures to deviate from a phenotype that was representative of the normal bronchial epithelium *in vivo*. Instead, the application of liquid caused dpHBEC cultures to exhibit a phenotype that was consistent with a variety of airway diseases, as well as the effects elicited by a broad range of inhaled substances that cause adverse effects in the respiratory tract. The presence of a disease phenotype can alter the response of the cultures following exposure to a test substance. For example, differentiated epithelial cultures derived from COPD patients express greater levels of IL-8 at baseline and in response to rhinovirus challenge compared to their normal, healthy counterparts^44,45^. Conversely, the induction of IL-8 following cigarette smoke exposure was lower in epithelial cultures isolated from COPD patients than cultures derived from normal, healthy donors^46^.

### Cell signaling

The response of the bronchial epithelium to inhaled toxicants and biological stimuli is mediated through the modulation of cellular signaling pathways, which regulate gene expression, cell survival and growth, and functional properties of the cell layer. Here, we observed that the application of liquid induces a significant increase in HIF-1a protein stabilization at 6 hours that is followed by increased p38 and ERK1 phosphorylation at 24 hours (Fig. 5). Activity of the HIF-1 signaling pathway is minimal under normoxic conditions in the healthy airway epithelium^24^. Under reduced oxygen concentrations, hypoxia-responsive gene expression is upregulated by the activation of the HIF-1 transcription factor following stabilization and nuclear translocation of its HIF-1a subunit^47^. Expression of HIF-1-responsive genes, including VEGF-A, ADM, and GLUT3, play key roles in angiogenesis, metabolism, and cell survival^48^. While the induction of HIF-1 signaling is often considered in the context of tumorigenesis/carcinogenesis, aberrant HIF-1a signaling also occurs in asthma, COPD, lung inflammation, acute lung injury, and ischemic lung injury^24,48–51^. HIF-1 activation also results from exposure to inhaled substances including cigarette smoke, particulate matter, and many natural product-derived small molecules^52–54^. HIF-1a activation also contributes to EMT and enhanced pro-survival signaling^55^.

ERK and p38 have diverse biological functions that include responding to cellular stress as well as promoting cell growth and survival^27,28,56,57^. The alternative regulation of cellular signaling proteins such as ERK1 and p38, alone or in concert, has been observed in response to exposures to a broad range of inhaled toxicants including ozone, diesel exhaust particulates, acrolein, asbestos, cigarette smoke, and diacetyl (2,3-butanedione)^58–63^. Further, the alternative regulation of these signaling proteins also occurs in a variety of respiratory diseases including COPD, asthma, fibrosis, EMT, and cancer^64–68^. Similar to HIF-1, the activation of these signaling proteins by liquid application alone has the potential to confound exposure outcomes and interpretation. For example, the activation of ERK and p38 by the application of liquid alone may have an additive or synergistic effect on the activation of these proteins, or other signaling pathways, that occurs as a result of exposure to a test substance. Thus, exposure to the test substance by liquid application could enhance toxicity-related exposure outcomes that are dependent on ERK and/or p38 activation such as the induction of pro-inflammatory gene expression in pHBEC cultures^58,69^. Alternatively, the role of HIF-1a, ERK, and/or p38 activation in pro-survival and growth signaling may mean that the activation of these pathways by the application of liquid alone could mitigate the cytotoxic effects of a test substance applied by liquid dosing. The pleiotropic effects of HIF-1a, ERK, and p38 provide many possible avenues by which the effect of liquid application could impact the outcome and/or interpretation of chemical testing studies that utilize liquid dosing of dpHBEC-ALI cultures.

### Induction of pro-inflammatory mediators

The airway epithelium plays a central role in the release of pro-inflammatory cytokines that play concerted roles in the response to inflammatory stimuli, airway insult/injury, and tissue homeostasis. While beneficial in the resolution of damage, their dysregulation results in airway remodeling, a chronic inflammatory state, impaired lung function, and disease^70^. Given the role of inflammation and response to tissue injury in the etiology of toxicant-induced effects, several pro-inflammatory cytokines, such as IL-8 and TNF-α, are common endpoints in the evaluation of chemical toxicity. In the study reported here, the application of liquid alone induced significant increases in the release of IL-8 and IL-1β at 6 hours and IL-8, IL-1β, and TNF-α at 24 hours (Fig. 6). The increased release of these pro-inflammatory cytokines has been implicated in a variety of respiratory diseases including COPD, acute lung injury, epithelial-to-mesenchymal transition, and squamous cell metaplasia^71–74^. Further, the dysregulation of pro-inflammatory cytokine secretion also results from exposure to a diverse range of inhaled substances including diesel exhaust particulates, ambient particulate matter, acrolein, cigarette smoke, e-cigarette flavorings, and zinc oxide nanoparticles^39,75–78^. Therefore, the observation that liquid application alone significantly enhances pro-inflammatory cytokine secretion indicates that the effects of this dosing method alone can induce a disease-like phenotype and could limit the identification of test substances that induce a pro-inflammatory response in the bronchial epithelium. Further, the induction of pro-inflammatory mediators by liquid application alone could account for the significantly lower levels of IL-8 induction in dpHBEC exposed to reactive aldehydes by liquid application dosing compared to matched ALI dosing reported by Dwivedi *et al*^79^. The increased secretion of pro-inflammatory mediators by the bronchial epithelium also has the potential to prime the culture to respond more strongly to test substance exposure compared to the epithelium at ALI or the *in vivo* airway. For example, HTB-54 lung epithelial cells primed with TNF-α, were found to secrete more IL-8 following particulate matter exposure compared to non-primed cells^80^. Additionally, the detection of elevated IL-8, TNF-α, or IL-1β is considered a biomarker for a wide variety of inflammatory conditions *in vivo*^81–83^. Thus, the use of liquid application dosing may invalidate the use of pro-inflammatory cytokine release as an *in vitro* biomarker that is directly linked to *in vivo* mediators of toxicity.

### Airway epithelial barrier integrity

The healthy bronchial epithelium functions as the barrier the protects the underlying lung tissue from inhaled pathogens, chemicals, and other materials. We observed that the application of liquid to dpHBEC significantly decreased barrier integrity and increased permeability as measured by a reduction in TEER and an increase in 20 kDa FITC-Dextran permeability at 24 hours, respectively (Fig. 7). Loss of epithelial barrier integrity is a hallmark of airway injury and occurs in airway diseases including COPD, acute respiratory distress syndrome, asthma, fibrosis, and EMT/cancer, thus supporting the conclusion that the application of liquid to dpHBEC causes a phenotype that is consistent with a range of respiratory diseases^41,84–87^. In contrast, hypoxia is protective against oxidant-induced loss of barrier integrity^88^; however, our observations indicate that the stabilization of HIF-1a protein alone (Fig. 5) is not sufficient to prevent the loss of barrier integrity following liquid application. Epithelial barrier integrity is also adversely affected following exposure to a diverse range of inhaled substances including diesel exhaust particulates, ambient particulate matter, acrolein, asbestos, ozone, wood smoke particulates, and e-cigarette flavorings^38–40,88–93^. Thus, the observation that liquid application alone significantly reduces barrier integrity indicates the potential for this manner of dosing to limit the sensitivity of this approach to identify test substances that disrupt barrier function.

The polarized nature of the respiratory epithelial barrier results on the segregation of receptors and other factors between their apical and basolateral surfaces, which is important in maintaining tissue homeostasis. Liquid-induced disruption of barrier integrity could result in aberrant distribution of both cell surface receptors and/or the test substance relative to exposures conducted under ALI conditions or by inhalation *in vivo*. For example, members of the human epidermal growth factor receptor (HER) family are differentially localized to the apical and basolateral compartments of the airway epithelium, which plays a critical role in their function *in vivo*^86^. Under homeostatic conditions, the epidermal growth factor receptor (EGFR; dimer of HER1) and the heterodimeric HER2/3 receptor are localized to the basolateral surface of the airway epithelium where they are separated from their ligands, epidermal growth factor (EGF) and amphiregulin, respectively, which are expressed in the apical compartment^94,95^. When this compartmentalization is disrupted by damage to the epithelium, EGF and heregulin are able to access their respective receptors to promote repair of the epithelium through cell proliferation, differentiation, and protection^86,96−98^. Further, members of the toll-like receptor (TLR) family are also selectively localized to the apical or basolateral surfaces of airway epithelial cells where they play distinct roles in the recognition of exogenous pathogen-associated molecular patterns (PAMPs) and endogenous damage-associated molecular patterns (DAMPs)^99,100^. Activation of the HER and TLR families are involved in various diseases of the respiratory tract and are activated in response to a wide range of inhaled chemicals/materials as well as DAMPs resulting from the effects of reactive oxygen species that are common mediators of inhaled toxicants^100–102^. Thus, loss of barrier integrity resulting from liquid application could result in the artificial exposure of test substances to receptors that they would not interact with under ALI conditions. Liquid application-induced decompartmentalization could also induce a pro-repair response that could limit the effects of test substances on cell survival. Further, activation of EGFR due to loss of epithelial barrier function also promotes mucus production^103^, which could be interpreted as an adverse effect of exposure and/or alter the interaction of the test substance with the epithelial cell surface. Importantly, these effects may not be easily accounted for by the normalization of the effects of test substance exposures to vehicle controls.

### Study limitations

The study reported here provides novel insight into the effects of liquid application on dpHBEC but has limitations that should be considered. First, we have previously shown that some endpoints vary based on inter-individual variability^69,92^. While the range of inter-individual variability for the endpoints evaluated here has not been evaluated, this study utilized an *n* of three for each endpoint evaluated, which may not fully reflect the range of variability in the effects of liquid application in the “normal healthy” donor population. Second, the liquid application dosing method has been used with a broad range of applied liquid volumes and in different permeable cell culture insert configurations (*e.g*., manufacturers, insert diameters, pore sizes, pore densities, membrane materials, *et cetera*). The observations reported here only evaluated one set of conditions. Third, different aqueous solvents including water, 0.9% saline, buffered (phosphate or other) saline, or cell culture medium have been used in reported studies that utilized liquid application dosing. Here we used 250 μL of ALI medium in 12 mm Corning Transwell^→^ inserts (223 μL/cm^2^; equal to 73.5 μL in a 6.5 mm Transwell insert), which was the smallest volume that we were able to use while maintaining complete coverage of the apical surface of the dpHBEC culture for the 24-hour treatment duration. We selected ALI medium as the applied liquid instead of PBS, 0.9% saline, or water to avoid generating an ionic gradient across the epithelial barrier and reducing nutrient availability. Additionally, while 0.9% saline is commonly used as a vehicle, it does not contain a pH buffer and has a broad pH range (manufacturer specification range of 4.5–7.0), which is often below pH 6.0 based on our evaluation of certificates of analysis from the commonly used manufacturers Baxter (#2F7123/#2F7124, lots #G154987, G139168, G160308, and G140409; mean pH 5.63 at room temperature according to certificates of analysis) and Cytiva (#Z1376, lots #WH30698322, WH30698323, WH30698324, and WH30698325; mean pH 5.79 at room temperature according to certificates of analysis).

The impact of the type, volume, and duration of liquid application on the effects observed in dpHBEC cultures should be evaluated in future studies to provide a more comprehensive understanding of these variables on *in vivo* physiology-relevant endpoints, exposure outcomes, and test substance delivery/dosimetry. While this study sought to describe the effects of liquid application on the dpHBEC-ALI system, future studies could also provide a thorough characterization of the molecular mechanisms underlying the effects reported here. More specifically, additional work could more comprehensively evaluate the hypotheses generated with the RNA-sequencing data regarding the alternative regulation of biological pathways by liquid application. Additional studies could also determine the relative contributions of the MAPK pathways and HIF1α signaling in the response of dpHBEC-ALI cultures to liquid application. Further studies are also required to determine whether differentiated primary human airway epithelial cell ALI models of other regions of the respiratory tract are similarly affected by the application of liquid alone.

## Conclusions

**Figure 8:**
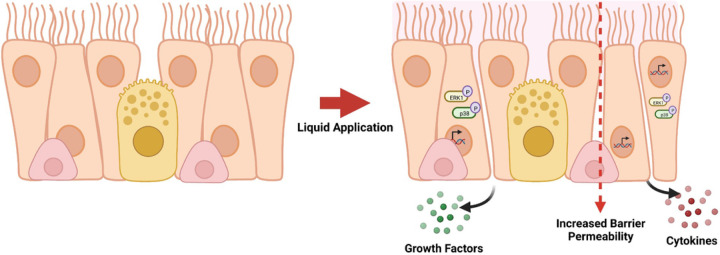
Summary of effects caused by liquid application to dpHBEC-ALI cultures. The application of 250 μL ALI medium induces the alternative regulation of transcripts, the activation of stress-responsive protein signaling pathways, the secretion of pro-inflammatory cytokines and growth factors, and the breakdown of epithelial barrier integrity.

*In vitro* studies that utilize differentiated primary human airway cell systems to evaluate inhalable test substances frequently rely on direct application dosing; however, publications describing these studies rarely report the use of an ALI control (*i.e*., not subjected to liquid application) to account for the effects of the application of liquid alone in the data analysis and interpretation. The findings described here demonstrate that the application of liquid alone can cause large-scale reprogramming of gene expression in differentiated ALI cultures and significant changes to aspects of the cell culture physiology that are of great importance with respect to the ability of the dpHBEC-ALI system to represent key aspects of *in vivo* respiratory tract physiology. Further, the changes that result from liquid application involve the alternative regulation of cellular pathways that are often involved in mediating the response to exogenous stimuli and diseases of the respiratory tract. The endpoints that we observed to be alternatively regulated by liquid application are also commonly used to identify and quantify the toxicity or efficacy of test substances (*i.e*., epithelial barrier function, cellular signaling pathway activation, pro-inflammatory cytokine release, and growth factor production) (Fig. 8). Additional studies are required to provide a better understanding of the comparability of liquid application and ALI dosing outcomes in differentiated primary human airway epithelial tissue models; however, the findings reported here support the conclusion that the impact of the application of liquid alone has the potential to confound the accuracy, sensitivity, and translation of data resulting from the use of liquid dosing of dpHBEC-ALI systems for respiratory biology research and the evaluation of inhaled substances.

The use of differentiated ALI cultures to inform inhaled chemical hazard identification and human health risk assessment is in its relative infancy, but the demand of the use of the approach is increasing. This is evidenced by the use of this dosing method in a recent case study for the use of an ALI-differentiated *in vitro* system as a part of an integrated approach to testing and assessment (IATA) to refine an inhalation risk assessment by the Organisation for Economic Co-operation and Development (OECD)^1,2^, an organization that develops international guidelines for studies used to evaluate pharmaceuticals, as well as commercial and industrial chemicals. Identification of considerations and limitations to the use of direct application dosing of ALI cultures at this early stage is critical for defining the context for their use in the assessment of inhalable substances. It also provides guidance on important aspects of study design and reporting, as well as considerations for the interpretation of data derived from liquid application dosing of dpHBEC-ALI systems, and potentially differentiated primary cell-based ALI models of other regions of the human respiratory tract. Additional studies are needed to determine whether the effect of liquid application alone impacts the outcomes of *in vitro* inhaled chemical testing, whether the effects observed here are specific to the conditions used in this study, and if these effects apply to differentiated ALI models of other regions of the human respiratory tract. Future studies are also needed to determine whether aspects of this dosing method (*e.g*., type of aqueous buffer used, liquid volume, exposure duration, *et cetera*) can be optimized to minimize the effects on culture physiology. Ultimately, improving our understanding of how key aspects of differentiated ALI models of the human respiratory tract impact their physiology, response to test substance exposures, and data analysis and interpretation is central to building confidence in their ability to reflect *in vivo* human outcomes and advancing the translation of data from these *in vitro* systems into decision making.

## Figures and Tables

**Table 1:** Summary of pro-inflammatory cytokine and growth factor ELISA data.

Analyte	ALI Concentration (pg/mL)	Concentration at 6H (pg/mL)	Fold Change at 6H (LAE/ALI)	*p* -value	Concentration at 24H (pg/mL)	Fold Change at 24H (LAE/ALI)	*p* -valui
IL-8	1338.00	2739.00	2.0	0.014	11820.00	8.8	0.012
IL-1β	1.13	2.23	2.0	0.027	7.41	6.6	0.001
IL-6	15.90	26.68	1.7	0.434	141.40	8.9	0.161
TNF-α	1.46	2.62	1.8	0.189	9.43	6.5	0.008
PDGF-B	0.79	0.70	0.9	0.883	2.16	2.7	0.385
PGF	6.01	5.42	0.9	0.817	58.56	9.7	0.025
VEGFA	1161.00	1119.00	1.0	0.532	3557.00	3.1	0.012

## Data Availability

All data included in the research manuscript are available from the primary author upon request. The differential gene expression results are publicly available in the NCBI Gene Expression Omnibus (series number: GSE198884). The data described in this manuscript are available upon publication at the Environmental Protection Agency’s online data repository EPA ScienceHub (https://catalog.data.gov/dataset/epa-sciencehub).
